# Association between weather features and symptoms in hand osteoarthritis: Results from the DIGICOD cohort

**DOI:** 10.1016/j.ocarto.2025.100639

**Published:** 2025-06-05

**Authors:** Mathilde Pezot, Romane Lacoste-Badie, Sophie Tuffet, Alexandra Rousseau, Pascal Richette, Bruno Fautrel, Francis Berenbaum, Alice Courties, Jérémie Sellam

**Affiliations:** a Department of Rheumatology, Saint-Antoine Hospital, Assistance Publique–Hôpitaux de Paris (AP-HP), Paris, France; b Sorbonne University, URCEST, CRB, CRC, Saint-Antoine Hospital, AP-HP, Paris, France; c Department of Rheumatology, Lariboisière Hospital, INSERM, Université de Paris, AP-HP, Paris U1132, France; d Department of Rheumatology, La Pitié-Salpetrière Hospital, INSERM UMRS, Sorbonne Université, AP-HP, Paris 1136, France; e Centre de Recherche Saint-Antoine (CRSA) Inserm UMRS-938, Sorbonne Université, Paris, France

**Keywords:** Osteoarthritis, Weather, Hand, Pain, Cohort

## Abstract

**Objective:**

This cross-sectional study aimed to investigate the association between weather and joint symptoms in patients with hand osteoarthritis (HOA).

**Design:**

We used baseline data from the DIGICOD cohort, a monocentric cohort of patients with HOA, and meteorological measurements (temperature, humidity, barometric pressure) in the Paris region during the 72 ​h before inclusion in the cohort. Clinical outcomes were AUSCAN subscores (pain, stiffness, function), spontaneous and pressure tender joint count (TJC), visual analog scale (VAS) score for hand pain during activity and at rest, and the Functional Index for Hand Osteoarthritis score. We used logistic regression models to search for associations between meteorological measurements and clinical outcomes, adjusting for sex, age, Kellgren-Lawrence score and Hospital Anxiety and Depression Scale score.

**Results:**

We analyzed data for 377 patients (mean age 66.5 ​± ​7.4 years, 85 ​% female). The AUSCAN-pain subscale score was not significantly associated with temperature, humidity or barometric pressure. Only spontaneous TJC was associated with relative humidity, and TJC at pressure was negatively associated with barometric pressure, both without dose-effect. Pain scores were not associated with temperature, and function and stiffness scores were not associated with any meteorological variable.

**Conclusions:**

This is the first study to investigate in a large cohort the association between meteorological factors and HOA symptoms. Despite a few isolated associations, our results do not clearly support the worsening of hand joint symptom during humid or rainy weather.

## 1 Introduction

At the present time, osteoarthritis (OA) management is based on preventive and symptomatic therapies. The main clinical symptom in OA is pain, which is a complex phenomenon, with multiple mechanisms (i.e., nociceptive, neuropathic and nociplastic) [1]. However, pain is the major symptom responsible for loss of quality of life in OA patients and drives many of the clinical decisions in therapeutic strategies [2]. Hence, we need to understand OA pain in order to help patients cope with it at best. Many factors affect OA-related pain such as sex, psychological states, and adiposity [3]. Environmental factors such as weather could be influential.

When patients are asked about their pain, many spontaneously report that pain can be influenced by the weather, especially during cold or rainy days [4,5]. This association of weather and rheumatic pain has been mentioned since ancient times: Hippocrates mentioned it in his treatise “On Airs, Waters and Places”. This “weather-sensitivity” is often mentioned in cultural works (literature, films etc.), and there are cases of notorious French people with joint diseases who moved to the south of France believing that a warmer climate could cure their illness. Two examples are the painters Pierre-Auguste Renoir and Raoul Dufy, who both had rheumatoid arthritis [6]. Today, the association is still a very well-established popular belief: in qualitative studies, patients frequently report that cold and damp weather increases their level of pain [7,8], and recent studies have shown that about two-thirds of patients with OA perceive the weather as affecting their pain [4,5]. The weather is now part of the Osteoarthritis Symptom Inventory Scale questionnaire, developed in 2023, to qualitatively assess pain in OA [9].

However, numerous studies have investigated the association between environmental weather and joint symptoms in OA and other rheumatic diseases, but their results are conflicting [10]. Although some results are in line with this popular opinion, showing exacerbated pain at low ambient temperatures [11,12] and high relative humidity [13,14], others suggest the opposite [15,16], and others found an association with a change in meteorological variables [11,17,18]. Moreover, some studies did not demonstrate any effect of weather [19]. The reasons for these discrepancies are mainly the differences in exposure time, outcomes, or adjustment factors (severity of OA, mood, medication consumption etc.) across studies. In addition, some studies included both OA and other rheumatic diseases, and most explored only a few patients [20,21]. Thus, the relation between weather variables and symptoms remains to be elucidated.

Among all the studies of OA, most investigated patients with knee or hip OA, but only one was dedicated to hand osteoarthritis (HOA) symptoms and included 32 patients. This study showed no association between symptoms and weather variables [21,22]. Overall, the effect of weather on symptoms in HOA is still unclear.

The first objective of this cross-sectional study was to determine whether some weather variables, namely temperature, relative humidity, or barometric pressure, are associated with pain in patients with HOA. Secondary objectives were to determine whether these weather factors were associated with joint stiffness or functional disability.

## 2 Methods

### 2.1 Study participants: the DIGICOD cohort

The DIGICOD, a monocentric hospital-based cohort, included 426 analyzable patients with symptomatic HOA between April 2013 and June 2017 from the rheumatology department of Saint-Antoine hospital (Paris). Its main objective was to investigate clinical, biological, genetic and imaging prognostic factors in HOA progression after 6 years of follow-up [23]. Patients were also recruited from other Paris university hospitals (Cochin, Pitié-Salpêtrière, Henri-Mondor hospitals), from general practitioners, private rheumatologists, or via public conferences or media advertisements.

Inclusion criteria were age >35 years and symptomatic HOA according to American College of Rheumatology criteria, defined as ≥ 2 symptomatic (pain or enlargement) joints among proximal or distal interphalangeal joints, thumb interphalangeal joint or first carpometacarpal joint. Patients had to have a radiological Kellgren-Lawrence score ≥2, be able to understand questionnaires in French and benefit from a social security system.

Exclusion criteria were the presence of another destructive rheumatic condition, a calcium pyrophosphate-crystal deposition disease or polyarticular gout involving hands, secondary OA related to traumatic or infectious disease, OA related to genetic cartilaginous dysplasia, and current pregnancy or breastfeeding. All patients gave their written informed consent. All patients of the DIGICOD cohort were followed for 6 years, during which demographic, clinical, biologic and radiographic information were collected.

The present study is a cross-sectional study based on the clinical database at the baseline visit.

### 2.2 Clinical outcomes

The primary outcome was the Australian/Canadian osteoarthritis hand index (AUSCAN)-pain subscale score at the inclusion visit [24]. Secondary outcomes were the AUSCAN-stiffness and -function subscale scores, visual analog scale score for hand pain during activity and at rest, spontaneous or pressure tender joint count (TJC), and Functional Index for Hand Osteoarthritis score [25] at the inclusion visit. The three AUSCAN subscales, the Functional Index for Hand Osteoarthritis score and the visual analog scale score for hand pain during activity were standardized on a scale from 0 to 100. For TJC, the 30 hand joints assessed were carpometacarpal, metacarpo-phalangeal and interphalangeal joints of both thumbs and metacarpophalangeal, distal and proximal interphalangeal joints of other fingers.

### 2.3 Meteorological data

We selected three meteorological variables: temperature, expressed in Celsius degrees (°C); relative humidity, expressed in percentage (%); and barometric pressure, expressed in hectopascals (hPa). From the Meteo-France website (https://donneespubliques.meteofrance.fr) (Meteo-France is France's official weather and climate service), we collected the values of these variables measured at Saint-Antoine Hospital's closest meteorological station, Orly (covering all Paris area; Global Positioning System coordinates: 48,72°N | 2,38°E) on the day of the inclusion visit and the two previous days for each patient. We chose a time period of 3 days, based on patients' experience and following the previously existing literature. In our study, we used these weather variables as their mean values over these three days.

### 2.4 Statistical analyses

We first describe patients’ general characteristics. Categorical variables are described with number (%) and quantitative variables with mean ​± ​SD if normally distributed or median (interquartile range and range) if non-normally distributed. Because the primary and secondary outcomes followed a non-normal distribution, they were redefined in binary form by their median value to define a high and low intensity of the symptoms.

The association between each weather variable and each outcome was first explored with an unadjusted logistic regression model. Then, each model was adjusted on age, sex, sum of Kellgren-Lawrence score for all joints (evaluated from 0 to 128), and Hospital Anxiety and Depression Scale (HADS) score [26]. Results are presented with odds ratios and 95 ​% confidence intervals. With the assumption of linearity between weather variables and the logit of the outcomes not verified, temperature, relative humidity and barometric pressure were introduced in the models as categorical variables by quartiles, the lower quartile being the reference modality. Analyses were conducted using SAS version 9.4. *P* values less than or equal to 0.05 were considered significant.

## 3 Results

### 3.1 General characteristics and meteorological measurements

From among the 426 patients included in the DIGICOD cohort, we excluded 49 (12 ​%) because of missing outcomes or adjustment factors, which left 377 patients (88 ​%) for the analysis. The characteristics of these patients are in Table 1.

**Table 1:** Characteristics of patients (n ​= ​377).

General characteristics	
Age at inclusion (years)	66.5 ​± ​7.4
Female sex	320 (84.9)
HADS score	12.6 ​± ​6.1
**HOA features**	
AUSCAN-pain subscale score (0–100)	20.0 (8.0–38.2)
AUSCAN-stiffness subscale score (0–100)	22.0 (7.0–53.0)
AUSCAN-function subscale score (0–100)	31.9 (13.2–55.3)
VAS score for hand pain during activity (0–100)	42.0 (22.0–66.0)
VAS score for hand pain at rest (0–100)	15.0 (0.0–35.0)
Spontaneous TJC score (0–30)	0.0 (0.0–2.0)
Pressure TJC score (0–30)	3.0 (2.0–6.0)
FIHOA score (0–100)	16.7 (3.3–33.3)
Kellgren-lawrence score (0–128)	46.2 ​± ​17.6

Data are mean ​± ​SD or median (IQR). AUSCAN: Australian/Canadian osteoarthritis hand index; FIHOA: Functional Index for Hand Osteoarthritis; HADS: Hospital Anxiety and Depression Scale; HOA: hand osteoarthritis; IQR: interquartile range; TJC: tender joint count; VAS: visual analog scale.

Weather variables (mean over the 3 days prior to inclusion) are described in Table 2. Median values were 11 ​°C (IQR: 6.2–15.3, range 2.5–28) for temperature, 79.3 ​% (68.7–85.6, range 43–96) for humidity and 1006.19 ​hPa (1000.94–1011.73, range 984.64–1022.97) for barometric pressure.

**Table 2:** Meteorological measurements from the Paris Orly station (mean over the 3 days before inclusion) (n ​= ​377).

**Temperature (°C)**	
***Median (IQR)***	11.0 (6.2–15.3)
***Quartiles, n (%)***	
1. [−2.5; 6]	85 (22.5)
2. [6; 11]	103 (27.3)
3. [11; 15]	84 (22.3)
4. [15; 28]	105 (27.9)
**Humidity (%)**	
***Median (IQR)***	79.3 (68.7–85.6)
***Quartiles, n (%)***	
1. [43; 68]	85 (22.5)
2. [68; 79]	100 (26.5)
3. [79; 85]	95 (25.2)
4. [85; 96]	97 (25.7)
**Barometric pressure (hPa)**	
***Median (IQR)***	1006.15 (1000.38–1012.79)
***Quartiles, n (%)***	
1. [984.64; 1000.94]	94 (24.9)
2. [1000.94; 1006.19]	93 (24.7)
3. [1006.19; 1011.73]	94 (24.9)
4. [1011.73; 1022.97]	96 (25.5)

IQR: interquartile range.

### 3.2 Association between temperature and symptoms

Results for adjusted and unadjusted models for the association between symptoms and temperature are in Table 3. Temperature was not associated with AUSCAN-pain subscale score (p ​= ​0.39 in the adjusted model) or any of the other secondary outcomes.

**Table 3:** Association between temperature and symptoms (logistic regression models).

Outcome	Unadjusted model		Model adjusted on age, sex, sum of Kellgren-Lawrence score, HADS score	
	OR (95 ​% CI)	p-value	OR (95 ​% CI)	p-value
**AUSCAN-pain subscale ≥ 20**				
***Temperature (°C)***		0.32		0.39
1. [−2.5; 6]	1		1	
2. [6; 11]	0.69 (0.38–1.23)		0.72 (0.40–1.30)	
3. [11; 15]	0.61 (0.33–1.12)		0.66 (0.35–1.23)	
4. [15; 28]	0.61 (0.34–1.09)		0.60 (0.33–1.09)	
**AUSCAN-stiffness subscale score ≥ 22**				
***Temperature (°C)***		0.80		0.73
1. [−2.5; 6]	1		1	
2. [6; 11]	1.13 (0.64–2.00)		1.40 (0.75–2.61)	
3. [11; 15]	0.93 (0.51–1.70)		1.18 (0.61–2.26)	
4. [15; 28]	1.22 (0.69–2.16)		1.33 (0.72–2.47)	
**AUSCAN-function subscale score ≥ 32**				
***Temperature (°C)***		0.54		0.73
1. [−2.5; 6]	1		1	
2. [6; 11]	0.88 (0.49–1.56)		0.93 (0.50–1.70)	
3. [11; 15]	0.73 (0.40–1.34)		0.81 (0.42–1.53)	
4. [15; 28]	1.11 (0.62–1.96)		1.14 (0.62–2.10)	
**At least 3 joints with pain at pressure**				
***Temperature (°C)***		0.46		0.50
1. [−2.5; 6]	1		1	
2. [6; 11]	0.71 (0.39–1.30)		0.76 (0.41–1.41)	
3. [11; 15]	0.84 (0.45–1.58)		0.95 (0.49–1.82)	
4. [15; 28]	0.63 (0.35–1.14)		0.66 (0.36–1.21)	
**At least 1 joint with spontaneous pain**				
***Temperature (°C)***		0.92		0.88
1. [−2.5; 6]	1		1	
2. [6; 11]	1.07 (0.60–1.90)		1.11 (0.62–1.98)	
3. [11; 15]	1.02 (0.56–1.87)		1.07 (0.58–1.97)	
4. [15; 28]	0.88 (0.50–1.57)		0.89 (0.50–1.60)	
**VAS score for hand pain during activity ≥ 42**				
***Temperature (°C)***		0.40		0.53
1. [−2.5; 6]	1		1	
2. [6; 11]	0.64 (0.36–1.13)		0.67 (0.37–1.22)	
3. [11; 15]	0.67 (0.36–1.23)		0.74 (0.39–1.38)	
4. [15; 28]	0.66 (0.37–1.18)		0.67 (0.37–1.22)	
**VAS score for hand pain at rest ≥ 15**				
***Temperature (°C)***		0.50		0.60
1. [−2.5; 6]	1		1	
2. [6; 11]	0.99 (0.56–1.75)		1.06 (0.59–1.91)	
3. [11; 15]	0.73 (0.40–1.34)		0.81 (0.43–1.50)	
4. [15; 28]	1.15 (0.65–2.04)		1.21 (0.67–2.18)	
**FIHOA score ≥ 17**				
***Temperature (°C)***		0.66		0.93
1. [−2.5; 6]	1		1	
2. [6; 11]	0.74 (0.41–1.32)		0.83 (0.44–1.54)	
3. [11; 15]	0.73 (0.40–1.34)		0.88 (0.46–1.69)	
4. [15; 28]	0.90 (0.51–1.60)		0.96 (0.52–1.78)	

AUSCAN: Australian/Canadian osteoarthritis hand index; FIHOA: Functional Index for Hand Osteoarthritis; OR: odds ratio; 95 ​% CI: 95 ​% confidence interval; VAS: visual analog scale.

### 3.3 Association between relative humidity and symptoms

The results of adjusted and unadjusted models for the association between symptoms and relative humidity are in Table 4. The AUSCAN-pain subscale score was not associated with relative humidity (p ​= ​0.14 in the adjusted model).

**Table 4:** Association between relative humidity and symptoms (logistic regression models).

Outcome	Unadjusted model		Model adjusted on age, sex, sum of Kellgren-Lawrence score, HADS score	
	OR (95 ​% CI)	p-value	OR (95 ​% CI)	p-value
**AUSCAN-pain subscale score ≥ 20**				
***Relative humidity (%)***		0.18		0.14
1. [43; 68]	1		1	
2. [68; 79]	1.61 (0.90–2.89)		1.76 (0.96–3.25)	
3. [79; 85]	1.88 (1.04–3.40)		1.99 (1.08–3.68)	
4. [85; 96]	1.34 (0.75–2.42)		1.52 (0.83–2.79)	
**AUSCAN-stiffness subscale score ≥ 22**				
***Relative humidity (%)***		0.52		0.33
1. [43; 68]	1		1	
2. [68; 79]	1.29 (0.72–2.31)		1.63 (0.85–3.12)	
3. [79; 85]	1.00 (0.56–1.79)		1.01 (0.53–1.90)	
4. [85; 96]	0.85 (0.47–1.51)		1.00 (0.53–1.88)	
**AUSCAN-function subscale score ≥ 32**				
***Relative humidity (%)***		0.89		0.97
1. [43; 68]	1		1	
2. [68; 79]	1.02 (0.57–1.81)		1.08 (0.58–2.02)	
3. [79; 85]	1.04 (0.58–1.87)		1.07 (0.58–2.00)	
4. [85; 96]	0.85 (0.47–1.51)		0.95 (0.51–1.76)	
**At least 3 joints with pain at pressure**				
***Relative humidity (%)***		0.28		0.23
1. [43; 68]	1		1	
2. [68; 79]	1.58 (0.88–2.85)		1.76 (0.95–3.28)	
3. [79; 85]	1.75 (0.96–3.20)		1.76 (0.95–3.28)	
4. [85; 96]	1.32 (0.73–2.38)		1.40 (0.76–2.56)	
**At least 1 joint with spontaneous pain**				
***Relative humidity (%)***		**0.02**		**0.02**
1. [43; 68]	1		1	
2. [68; 79]	1.83 (1.00–3.34)		1.74 (0.94–3.21)	
3. [79; 85]	2.71 (1.47–4.99)		**2.67 (1.44**–**4.94)**	
4. [85; 96]	1.94 (1.06–3.55)		**1.95 (1.06**–**3.61)**	
**VAS score for hand pain during activity ≥ 42**				
***Relative humidity (%)***		0.19		0.18
1. [43; 68]	1		1	
2. [68; 79]	1.89 (1.05–3.40)		**1.97 (1.06**–**3.63)**	
3. [79; 85]	1.59 (0.88–2.87)		1.58 (0.86–2.91)	
4. [85; 96]	1.46 (0.81–2.62)		1.56 (0.85–2.86)	
**VAS score for hand pain at rest ≥ 15**				
***Relative humidity (%)***		0.56		0.65
1. [43; 68]	1		1	
2. [68; 79]	1.12 (0.63–1.99)		1.13 (0.62–2.07)	
3. [79; 85]	1.41 (0.79–2.54)		1.39 (0.76–2.54)	
4. [85; 96]	0.97 (0.54–1.73)		1.00 (0.55–1.81)	
**FIHOA score ≥ 17**				
***Relative humidity (%)***		0.85		0.51
1. [43; 68]	1		1	
2. [68; 79]	1.21 (0.67–2.16)		1.42 (0.75–2.71)	
3. [79; 85]	0.95 (0.52–1.72)		0.94 (0.49–1.78)	
4. [85; 96]	1.08 (0.60–1.95)		1.29 (0.68–2.44)	

AUSCAN: Australian/Canadian osteoarthritis hand index; FIHOA: Functional Index for Hand Osteoarthritis; OR: odds ratio; 95 ​% CI: 95 ​% confidence interval; VAS: visual analog scale.

Spontaneous TJC was significantly associated with humidity in both unadjusted and adjusted models; the probability was increased with humidity >80 ​% (p ​= ​0.02 for both models) but without a dose effect: the OR was 2.67 (1.44–4.94) for humidity 79 ​%–85 ​% (the strongest association) and 1.95 (1.06–3.61) for humidity 85 ​%–96 ​% versus <68 ​%. No other secondary outcome was associated with relative humidity.

### 3.4 Association between barometric pressure and symptoms

Results for adjusted and unadjusted models for the association between symptoms and barometric pressure are in Table 5. The AUSCAN-pain subscale, again, was not associated with barometric pressure (p ​= ​0.78 in the adjusted model).

**Table 5:** Association between barometric pressure and symptoms (logistic regression models).

Outcome	Unadjusted model		Model adjusted on age, sex, sum of Kellgren-Lawrence score, HADS score	
	OR (95 ​% CI)	p-value	OR (95 ​% CI)	p-value
**AUSCAN-pain subscale ≥ 20**				
***Pressure (hPa)***		0.67		0.78
1. [984.64; 1000.94]	1		1	
2. [1000.94; 1006.19]	1.02 (0.58–1.82)		1.10 (0.61–1.99)	
3. [1006.19; 1011.73]	0.74 (0.42–1.32)		0.81 (0.45–1.46)	
4. [1011.73; 1022.97]	0.96 (0.54–1.69)		0.97 (0.54–1.74)	
**AUSCAN-stiffness subscale ≥ 22**				
***Pressure (hPa)***		0.38		0.17
1. [984.64; 1000.94]	1		1	
2. [1000.94; 1006.19]	0.82 (0.46–1.46)		0.97 (0.52–1.83)	
3. [1006.19; 1011.73]	1.35 (0.76–2.40)		1.73 (0.92–3.24)	
4. [1011.73; 1022.97]	0.92 (0.52–1.62)		0.92 (0.50–1.71)	
**AUSCAN-function subscale ≥ 32**				
***Pressure (hPa)***		0.84		0.92
1. [984.64; 1000.94]	1		1	
2. [1000.94; 1006.19]	1.02 (0.58–1.81)		1.11 (0.60–2.04)	
3. [1006.19; 1011.73]	0.81 (0.46–1.43)		0.90 (0.49–1.65)	
4. [1011.73; 1022.97]	1.00 (0.57–1.76)		1.03 (0.56–1.88)	
**At least 3 joints with pain at pressure**				
***Pressure (hPa)***		0.01		0.01
1. [984.64; 1000.94]	1		1	
2. [1000.94; 1006.19]	0.63 (0.34–1.17)		0.66 (0.35–1.26)	
3. [1006.19; 1011.73]	0.38 (0.21–0.70)		0.39 (0.21–0.74)	
4. [1011.73; 1022.97]	0.47 (0.25–0.86)		0.43 (0.23–0.81)	
**At least 1 joint with spontaneous pain**				
***Pressure (hPa)***		0.18		0.20
1. [984.64; 1000.94]	1		1	
2. [1000.94; 1006.19]	0.76 (0.43–1.35)		0.75 (0.42–1.34)	
3. [1006.19; 1011.73]	0.57 (0.32–1.02)		0.59 (0.33–1.06)	
4. [1011.73; 1022.97]	0.58 (0.32–1.03)		0.57 (0.32–1.01)	
**VAS score for hand pain during activity ≥ 42**				
***Pressure (hPa)***		0.46		0.39
1. [984.64; 1000.94]	1		1	
2. [1000.94; 1006.19]	0.94 (0.53–1.67)		0.98 (0.54–1.79)	
3. [1006.19; 1011.73]	0.77 (0.44–1.37)		0.85 (0.47–1.54)	
4. [1011.73; 1022.97]	0.66 (0.37–1.16)		0.63 (0.35–1.14)	
**VAS score for hand pain at rest ≥ 15**				
***Pressure (hPa)***		0.54		0.44
1. [984.64; 1000.94]	1		1	
2. [1000.94; 1006.19]	1.51 (0.85–2.68)		1.63 (0.90–2.96)	
3. [1006.19; 1011.73]	1.09 (0.61–1.93)		1.21 (0.67–2.17)	
4. [1011.73; 1022.97]	1.18 (0.67–2.09)		1.18 (0.66–2.11)	
**FIHOA score ≥ 17**				
***Pressure (hPa)***		0.55		0.45
1. [984.64; 1000.94]	1		1	
2. [1000.94; 1006.19]	1.11 (0.63–1.98)		1.36 (0.72–2.54)	
3. [1006.19; 1011.73]	0.74 (0.41–1.32)		0.86 (0.46–1.60)	
4. [1011.73; 1022.97]	0.88 (0.50–1.57)		0.87 (0.47–1.61)	

AUSCAN: Australian/Canadian osteoarthritis hand index; FIHOA: Functional Index for Hand Osteoarthritis; OR: odds ratio; 95 ​% CI: 95 ​% confidence interval; VAS: visual analog scale.

Pressure TJC was inversely associated with barometric pressure (p ​= ​0.01); that is, a decrease in barometric pressure was associated with more symptoms. With barometric pressure 10006.19 to 1011.73 ​hPa, the OR was 0.39 (0.21–0.74), and with barometric pressure 1011.73–1022.97 ​hPa, the OR was 0.43 (0.23–0.81) as compared with barometric pressure <1000.94 ​hPa. No other secondary outcome was associated with barometric pressure.

## 4 Discussion

In this cross-sectional study of patients with HOA, we found no significant association between our primary outcome, the AUSCAN-pain subscale, and any of the three meteorological variables. Similarly, neither hand function nor hand stiffness scores were associated with temperature, relative humidity, or barometric pressure. However, we observed isolated associations between joint tenderness and some weather features: over the previous 3 days, increased spontaneous TJC was associated with increased relative humidity, and increased pressure TJC was associated with reduced barometric pressure.

These results do not support the patients' beliefs: we found no strong evidence of worsening of joint pain according to the weather. Most of the “weather-sensitive” patients consider that their symptoms worsen during cold and rainy days; and so we could have expected higher joint symptoms scores with higher relative humidity or lower temperatures. Here, we found some associations between pain and high relative humidity and low barometric pressure: in fact, a rising relative humidity, associated with a drop in barometric pressure, are meteorological features associated with unstable weather and precipitations. Nevertheless, we did not find any association with colder temperatures, and the effect of relative humidity and barometric pressure are not statistically significant and are not dose-dependent. For example, spontaneous TJC was associated with mild and moderate relative humidity (second and third quartiles versus the first quartile), but this effect disappeared with higher humidity. A stronger relationship between symptoms and weather variables needs to be shown to support patients’ beliefs: as there may be tiny variations in weather variables in a well-tempered climate such as in Paris, we could hypothesize that a different kind of climate, with greater variations in meteorological variables, could have highlighted a stronger association between weather and symptoms.

To our knowledge, this is the largest cohort of HOA patients for which the association between weather and symptoms has been specifically studied. De Figueireido et al. [22] conducted a study of a small sample of 32 patients in Brazil in 2013: the SACRAH questionnaire was administered three times a week for 2 months, July and November, to assess the clinical symptoms pain, function and stiffness of HOA. The climate variables were compared to the evaluation of symptoms in each patient. However, the authors did not show any statistically significant correlation with individual variations in perceptions of pain. Such a small sample size was probably insufficient to show any robust association [22], but even with a larger cohort of patients, we did not found any statistically significant effect either. Among the other studies published on the association of weather and OA symptoms, few explored patients with HOA and mostly included patients with hip or knee OA. Some previous studies showed an association of symptoms with relative humidity: in 2015, an 810-patient cohort with OA including 443 with HOA found an association between humidity and pain for all patients regardless of age, sex, country of residence or HADS score; this effect was more pronounced with cold temperatures [13]. In 2014, a cohort study of 222 patients with hip OA highlighted a risk of increased Western Ontario and McMaster Universities Osteoarthritis Index (WOMAC) pain score with increased relative humidity [14]. Three other studies of various patients with OA and other rheumatic diseases found a significant association between increased pain scores and increased humidity [12,15,27]. However, in all these studies, the association of the weather and the symptoms remained very modest: for example, Dorleijn et al. [14] estimated that for every 10 ​% increase in relative humidity, the WOMAC pain score increased by 1 on a scale of 0–100. Only one study of patients with OA and rheumatoid arthritis found an association of pain and decreased barometric pressure [28]. In addition, several studies did not find any association with weather variables [19,29]; among the positives studies, several found an association of pain with some of the most frequently reported weather parameters (temperature, humidity, barometric pressure) but never the three of them. Two recent meta-analyses exploring this topic were published lately: first, a 2023 systematic literature review of 14 studies of weather and pain symptoms in OA found positive results in 13 of the studies [21]. A meta-analysis of five of these studies showed a negative association between pain and temperature and a positive association of pain with barometric pressure and relative humidity. Second, a 2024 meta-analysis of 3 case-crossover studies of weather and OA pain, showed with pooled analyses of that relative humidity, barometric pressure or temperature are not associated to a higher risk of OA pain or flare-up [10]. Hence, the relationship between weather and OA pain remains a conflicting topic. Despite some isolated associations between joint tenderness and some meteorological features, our study supports the fact that weather does not appear to frankly influence symptoms in a significant way.

This study has several strengths. The DIGICOD cohort is one of the largest cohorts of patients with HOA, and we included an important number of patients in this study. In contrast to several other studies conducted on this topic, all patients included in this study had the same disease, HOA. Therefore, these results cannot be generalized to all patients with OA, but this recruitment ensures a homogenous population of patients. Patients were recruited from several medical centers in Paris, from public and private practice and from primary, secondary or third health care centers, thus maintaining diversity in socio-economic status or socio-cultural beliefs, which could have been a confounding factor [4]. Although the monocentric design of the study implies that all patients were exposed to the same climate without extreme variations, it allowed for a standardized clinical and radiographic assessment of patients, with data obtained from a single meteorological center. The association of weather and the mood of patients represents a major confounding factor [30], given that the mood itself can exacerbate or diminish the joint pain [31]. For this reason, we adjusted our analyses on HADS score to better interpret the results. Even with this adjustment, the association between weather and some symptoms were significant, which suggests a link between weather and symptoms regardless of the mood. Finally, one of the potential confounding factors in studies exploring this association is confirmation bias: patients could be more likely to find associations between their symptoms and bad weather, although this could be a coincidence, because it comforts them in an idea that they have always believed in. However, this study was conducted retrospectively: patients were masked to its aim, which could have prevented them from reporting higher pain scores during rainy days.

There are several limitations to this study. First, in the absence of a clear pathophysiological explanation for weather sensitivity, we do not consider how long it might take for the weather to affect symptoms and how long they might persist. As a result, the methodological choices (such as the exposure time or symptom assessment time) can differ across studies on this topic, including ours, which could partly explain the discrepancies. In our study, we explored the weather 3 days before the symptom assessment, based on the patients' experience, but the possible association of the weather and joint pain could be faster or longer. Second, it is tough to get an accurate evaluation of patients' meteorological exposure: for example, we do not have information about the time spent indoors and outdoors, or the use of air conditioning. Patients may have stayed indoors longer during very bad weather, thus reducing their exposure to the ambient atmosphere and limiting its effect on symptoms. However, this is not true for atmospheric pressure, which is the same indoors and outdoors. Dixon and al [32]. evaluated chronic pain with a 2658 patient cohort over a 15-month period, using Global Positioning System on the participants’ smartphones. They found out that OA pain was increased with humidity, but also that time spent outside did not significantly modify the results. Third, we included only patients from the Paris area, based on the Saint-Antoine hospital location, but the inclusion of patients from other regions in France with a different type of climate could highlight a stronger association between symptoms and weather. At last, because patients do not often report the influence of sudden weather changes, we did not examine the variation in weather variables in the days preceding the symptoms, rather than their absolute values, although some authors have found an association with increasing pain [11,17]. The small associations we found between TJC at pressure and atmospheric pressure, and spontaneous TJC and relative humidity, might be furthered explored considering dynamic changes of the weather. We also limited the meteorological variables to temperature, relative humidity, and atmospheric pressure and not other variables such as sunshine, length of days, precipitation, and wind speed. However, the three selected variables are the most frequently reported factors associated with symptoms in other studies [21].

In conclusion, this study provides the first analysis of the association between HOA joint pain symptoms and weather in a large cohort and does not support a meaningful impact of weather on HOA pain. Further explorations are needed to understand the origins of the very popular idea of impact of weather on OA pain and its pathophysiological mechanisms.

## Author contributions

Conception and design, drafting of the article: M. Pezot and J. Sellam. Statistical expertise: R. Lacoste-Badie, S. Tuffet, A. Rousseau. Analysis and interpretation of the data, revision and final approval of the article: All authors.

## Ethics

The DIGICOD study complies with the Declaration of Helsinki and obtained all the regulatory and ethics validation from the local regulatory committee (Comité de Protection des Personnes, Paris Île-de-France IV).

## Data availability

Data sharing will be subject to the terms of DIGICOD cohort and the Assistance Publique–Hôpitaux de Paris data-sharing agreement to ensure all users of the data adhere to the legal requirements of using personal data. Meteorological data are free public information and were obtained from Météo-France website.

## Role of the funding source

The DIGICOD cohort was supported by the French Society of Rheumatology and an unrestricted grant from TRB Chemedica, which did not take part in the study design, collection, analysis, and interpretation of data, writing of the report or the decision to submit the article for publication.

## Declaration of competing interest

All authors have completed the ICMJE uniform disclosure form at www.icmje.org/coi_disclosure.pdf. AC received payment or honoraria for lectures, presentations, speakers bureaus, manuscript writing or educational events from Pfizer, UCB, Abbvie, Lilly, Janssen, and a Support for attending meetings and/or travel from Galapagos, Pfizer, BMS, UCB, MSD, Biogen, Novartis, Janssen. FB received consulting fees from Grunenthal, GSK, Eli Lilly, Novartis, Pfizer, Servier, 4P Pharma ; received payment or honoraria for lectures, presentations, speakers bureaus, manuscript writing or educational events from Viatris, Pfizer, Zoetis ; received support for attending meetings and/or travel from Nordic Pharma ; has patents planned, issued or pending with 4Moving Biotech, participated on a Data Safety Monitoring Board or Advisory Board with AstraZeneca, Sun Pharma, Nordic Bioscience ; stock or stock options with 4P Pharma, 4Moving Biotech. JS received consulting fees from Grunenthal, Pfizer, Jansser, Novartis, AlfaSigma, Abbvie, BMS, Lilly, UCB, MSD, Fresenius Kabi ; received payment or honoraria from MSD, Janssen, Celltrion, Fresenius Kabi, Pfizer, and received support for attending 1 meeting and/or travel from Janssen, Novartis, UCB. Other authors declare: no support from any organisation for the submitted work; no financial relationships with any organisations that might have an interest in the submitted work in the previous 3 years; no other relationships or activities that could appear to have influenced the submitted work.
